# Depletion of chondrocyte primary cilia reduces the compressive modulus of articular cartilage^☆^

**DOI:** 10.1016/j.jbiomech.2013.11.040

**Published:** 2014-01-22

**Authors:** Jerome Irianto, Girish Ramaswamy, Rosa Serra, Martin M. Knight

**Affiliations:** a Institute of Bioengineering and School of Engineering and Materials Science, Queen Mary University of London, Mile End Rd, London E1 4NS, United Kingdom; b Arthritis & Musculoskeletal Diseases Center, UAB School of Medicine and Dentistry, Birmingham, AL, USA; c Department of Orthopedic Surgery, Perelman School of Medicine, University of Pennsylvania, PA, USA

**Keywords:** Primary cilia, Chondrocyte, Cartilage, Biomechanics

## Abstract

Primary cilia are slender, microtubule based structures found in the majority of cell types with one cilium per cell. In articular cartilage, primary cilia are required for chondrocyte mechanotransduction and the development of healthy tissue. Loss of primary cilia in *Col2aCre;ift88*^*fl/fl*^ transgenic mice results in up-regulation of osteoarthritic (OA) markers and development of OA like cartilage with greater thickness and reduced mechanical stiffness. However no previous studies have examined whether loss of primary cilia influences the intrinsic mechanical properties of articular cartilage matrix in the form of the modulus or just the structural properties of the tissue. The present study describes a modified analytical model to derive the viscoelastic moduli based on previous experimental indentation data. Results show that the increased thickness of the articular cartilage in the *Col2aCre;ift88*^*fl/fl*^ transgenic mice is associated with a reduction in both the instantaneous and equilibrium moduli at indentation strains of greater than 20%. This reveals that the loss of primary cilia causes a significant reduction in the mechanical properties of cartilage particularly in the deeper zones and possibly the underlying bone. This is consistent with histological analysis and confirms the importance of primary cilia in the development of a mechanically functional articular cartilage.

## 1 Introduction

The primary cilium is a single cytoskeletal organelle that, in most cells, projects into the extracellular environment. It is composed of a characteristic array of microtubule doublets which are assembled by a process of intraflagellar transport (IFT) (Satir et al., 2010). The primary cilium functions as a signaling hub for an expanding range of pathways including hedgehog signaling, wnt signaling and mechanotransduction (for review see Berbari and O'Connor, 2009, Satir and Pedersen, 2010). Articular chondrocytes express primary cilia, which are typically 1–2 µm in length in situ, but longer in isolated cells in 2D culture (Wilsman, 1978, Poole and Jensen, 1997, Jensen and Poole, 2004, McGlashan and Cluett, 2008, Farnum and Wilsman, 2011). Recent studies have shown that chondrocyte primary cilia are required for mechanotransduction and associated up-regulation of extracellular matrix synthesis (Wann et al., 2012). Cilia are also involved in the response of chondrocytes to inflammatory cytokines (Wann and Knight, 2012) and the development of osteoarthritis (OA) associated with aberrant hedgehog signaling (Kaushik and Martin, 2009, Lin and Seeto, 2009). Recent studies from Serra’s group investigated the role of primary cilia in the development of articular cartilage (Chang et al., 2012) and growth plate (Chang and Serra, 2013). Studies used *Col2aCre;ift88*^*fl/fl*^ transgenic mice in which the chondrocytes lack primary cilia resulting in increased expression of OA markers including MMP13, ADAMTS5, COLX and RUNX2 (Chang et al., 2012) with changes in both the cartilage and the underlying bone. The study also attempted to characterize the mechanical properties of the articular cartilage in mutant and wild type mice using microindentation. However, although there was a reduction in stiffness for the *Col2aCre;ift88*^*fl/fl*^ cartilage, this was associated with a significant increase in tissue thickness. Thus, it was not possible to use the stiffness measurements as an indication of material properties of the cartilage, namely the modulus. In the current study we reanalyze the raw data to derive the viscoelastic moduli based upon the analytical models of Hayes et al. (1972) and Zhang et al. (1997). In so doing, we present a method for deriving cartilage moduli values from microindentation and show that loss of primary cilia reduces the moduli of articular cartilage, particularly in the deep zone. This correlates with alterations in the histology of the cartilage from wild type and *Col2aCre;ift88*^*fl/fl*^ mice. The study adds further evidence demonstrating the importance of chondrocyte primary cilia in the development of articular cartilage.

## 2 Materials and methods

### 2.1 Mechanical testing of cartilage

**Fig. 1:**
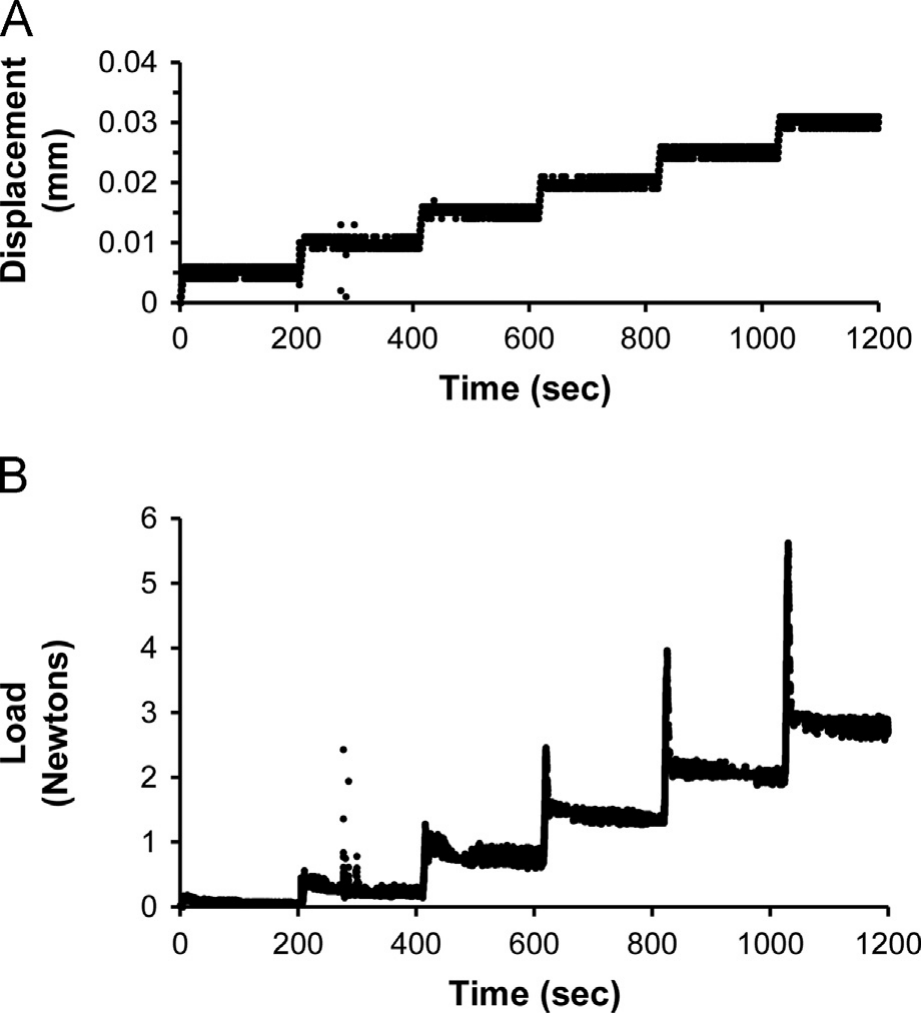
Representative plots showing indentation versus time (A) and force versus time (B). This original raw data from Chang et al. (2012) was used here to calculate the instantaneous and equilibrium moduli at each strain increment. A total of six displacement increments were applied creating a maximum indentation of 0.03 mm (30 µm) which corresponds to a strain of 57% for this sample with a thickness of 53 µm.

Chang, Ramaswamy et al. (2012) used microindentation to examine the mechanical properties of cartilage from 2 month old *Col2aCre;ift88*^*fl/fl*^ transgenic mice and wild type controls. To review briefly, tibia were mounted in bone cement and the articular cartilage on the tibial plateau was indented using a computer controlled electromechanical test system (Bose LM1), with a 200 g load cell (Sensotec). A plane ended impermeable cylindrical indenter (178 µm diameter) was advanced onto the tissue with a tare load of 0.05 g held for 200 s. Indentation was then applied in increments of 5 µm with 200 s relaxation time between each increment. Typical displacement and force versus time data is shown in Fig. 1. Following testing, the cartilage thickness was measured using the needle indentation method.

### 2.2 Moduli calculation

For each 5 µm displacement increment, the instantaneous modulus was calculated from the instantaneous or peak load and the equilibrium modulus was calculated from the subsequent minimum load at equilibrium. Previous studies by Hayes et al. (1972) have shown theoretically that the relationship between indentation load (P), indenter displacement ($\omega_{0}$), indenter radius (a), Poisson’s ratio (ν) and shear modulus (G) is determined by a scaling factor (κ), as shown by the following equation:

(1) $$\kappa =\frac{P(1-\nu )}{4aG\omega_{0}}$$

The value for κ is dependent on the ratio between the indenter radius and the cartilage thickness (a/h).

Meanwhile, Young’s modulus (E) can be calculated from the shear modulus by using the following equation:

(2) E=2G(1+ν)

Substituting Eqs. (1) and (2), Young’s modulus can be determined from the indentation results with the following equation:

(3) $$E=\frac{P(1-\nu^{2})}{2a\kappa \omega_{0}}$$

However, Hayes’s solution is based on two limiting assumptions. First, it was derived for a linear single phase material, making it not suitable for non-linear materials, including cartilage tissue. Second, Hayes’s solution also assumed very small deformation, typically an indentation strain of 0.1%. This is not suitable for most of indentation studies on cartilage, including the present study, where the strain is often larger than 0.1% due to the precision of the displacement control and irregularities in the tissue surface.

In order to overcome these limiting assumptions, Zhang et al. (1997) used a finite element (FE) computational modeling approach, to provide a new sets of κ values which are corrected for the non-linearity behavior of cartilage tissue upon larger deformations. It is important to note that the a/h ratios considered in Zhang’s study were between 0.2 and 2. This condition is met in the data from Chang et al. which is analyzed in this study. In addition, the strains considered in Zhang’s finite element model are between 0.1 and 15%. However, they state that the κ value is approximately proportional to the indentation strain and hence the κ values for the larger strains used in this study can be deduced by a linear interpolation of Zhang’s data (Zhang , 1997).

Therefore we have calculated the moduli values from the original indenter load and displacement data from Chang et al. (2012) using the solution from Hayes et al. (1972) with a modified scaling factor derived from Zhang et al. (1997). Due to the incompressibility nature of cartilage tissue upon the higher rate of loading, Poisson’s ratio was assumed to be 0.2 based on previous values reported for murine cartilage (Cao et al., 2006).

## 3 Results

**Fig. 2:**
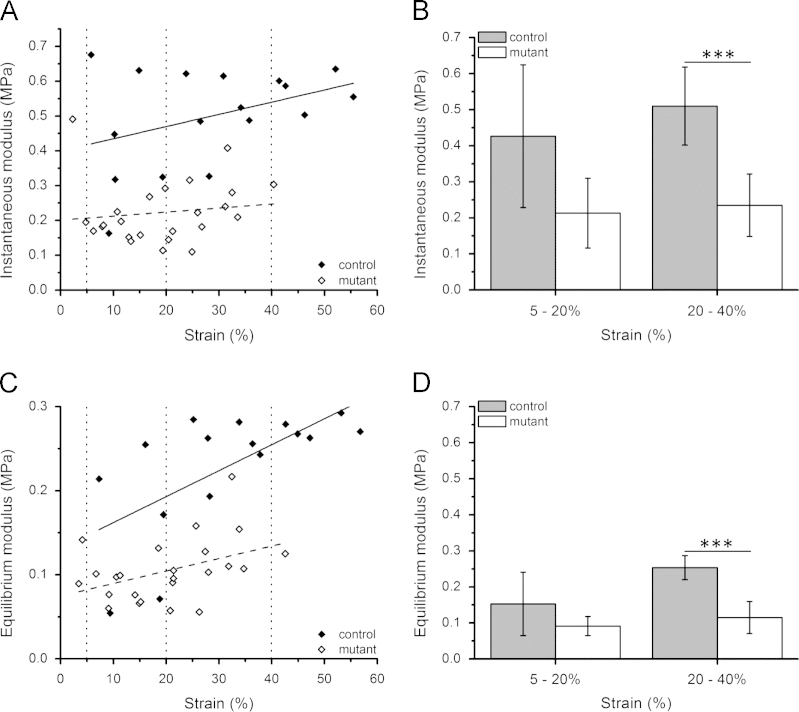
Instantaneous (A and B) and equilibrium moduli (C and D) calculated for articular cartilage from wild type (control) and *Col2aCre;ift88*^*fl/fl*^ transgenic mice (mutant) subjected to microindentation, with Poisson’s ratio of 0.2. Scatter plots (A and C) show all data points whilst the histograms (B and D) indicate the mean values for data grouped within the range of 5–20% strain (*n*=5–6 control, 9–11 mutant) and 20–40% strain (*n*=6 control, 10–12 mutant). Error bars indicating standard deviations. Data taken from a total of 34 measurements from 2 to 3 separate animals per group. Statistically significant differences are indicated at *p*<0.001 (^⁎⁎⁎^).

Displacement values were converted to strain based on the tissue thickness. The instantaneous and equilibrium moduli values were successfully calculated at each loading increment using the method described above. There were significant positive correlations between the moduli and the indentation strain for both *Col2aCre;ift88*^*fl/fl*^ mutant and control cartilage (Fig. 2). Moduli values were grouped according to the level of applied indentation strain, namely 5–20% and 20–40%. Values at strains above 40% were discarded due to the large mechanical contribution of the underlying bone and the lack of data for mutant mice (Fig. 2A and C). The *Col2aCre;ift88*^*fl/fl*^ mutant cartilage had lower instantaneous and equilibrium moduli, approximately half that seen for wild type control cartilage, although was only statistically significant at the higher level of indentation (20–40%, *p*<0.001) (Fig. 2B and D).

These studies used a Poisson’s ratio of 0.2. A sensitivity analysis over a range of Poisson’s ratio previously used for cartilage (Jurvelin and Buschmann, 1997, Wong and Ponticiello, 2000) showed that increasing the ratio reduced the moduli. The effect was greater for the thinner cartilage from wild type mice where an increase in Poisson’s ratio from 0.1 to 0.5 produced an increase in instantaneous and equilibrium moduli of approximately 0.2 MPa and 0.075 MPa respectively. However for all values of Poisson’s ratio the moduli calculated for cartilage from *Col2aCre;ift88*^*fl/fl*^ mutant mice were lower than those for wild type controls (data not shown).

## 4 Discussion

**Fig. 3:**
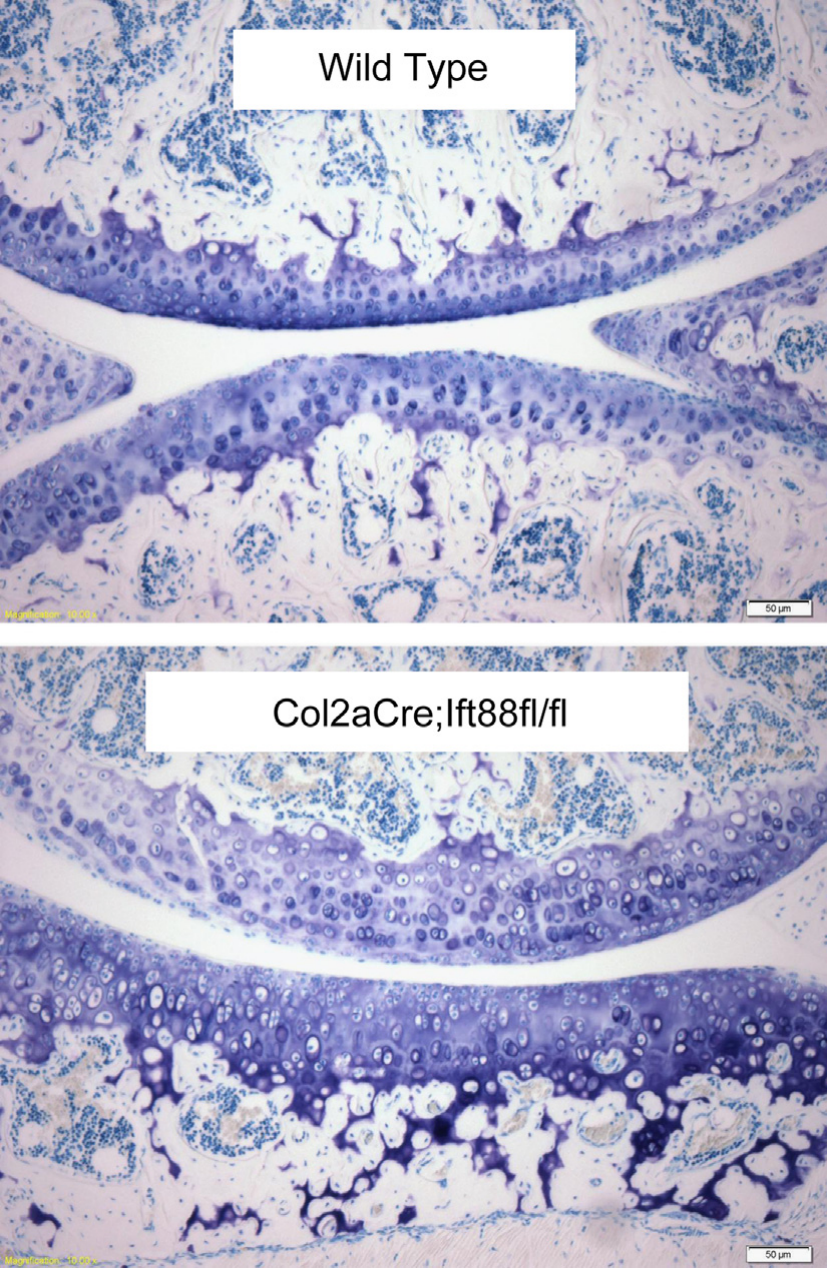
Histological sections stained with Toluidine blue showing abnormal joint formation in 2-month-old *Col2aCre;ift88*^*fl/fl*^ transgenic mice compared with wild type controls. The mutants have thicker cartilage with abnormal cell morphology, particularly in the deeper zones. Scale bar represents 50 µm.

Moduli values for wild type murine tibial cartilage are similar to those previous reported for articular cartilage with equilibrium moduli in the range of 0.1–0.5 MPa (Schinagl and Gurskis, 1997, Korhonen and Laasanen, 2002, Simha and Jin, 2007, Julkunen and Harjula, 2009). However other studies report elastic modulus for murine cartilage varying from 2 MPa based on microindentation (Cao et al., 2006) to 0.05 MPa based on AFM (Christensen et al., 2012). The increase in equilibrium moduli with indentation strain reflects the increased contribution of the stiffer underlying bone and the inherent inhomogeneity of the cartilage with the deeper zone tissue having a higher modulus. Loss of IFT88 and associated depletion of primary cilia in articular cartilage of *Col2aCre;ift88*^*fl/fl*^ mice resulted in a reduction in cartilage moduli although this only reached statistical significance at indentation of 20–40% (Fig. 2). This agrees with the histology data, which shows that loss of ift88 prevents normal apoptosis, particularly in the deep and calcified zones leading to thickening of the cartilage and abnormal joint formation in *Col2aCre;ift88*^*fl/fl*^ mice (Fig. 3) (Chang et al., 2012). Similar findings have also been reported using a IFT88 hypomorph model in which there is reduced chondrocyte hypertrophy in the developing growth plate (McGlashan et al., 2007). These results show that primary cilia are essential for the development of articular cartilage and the formation of a mechanically robust extracellular matrix.

## Conflict of interest statement

None of the authors have any conflicts of interest with regard to the above paper.
